# A Novel Zinc (II) Porphyrin Is Synergistic with PEV2 Bacteriophage against *Pseudomonas aeruginosa* Infections

**DOI:** 10.3390/antibiotics12040735

**Published:** 2023-04-10

**Authors:** Jessica Geyer, Kristen A. Krupa, Zachary M. Harris, Ying Sun, Lokesh Sharma, Silvia Würstle, Buqu Hu, Gail Stanley, Govindarajan Rajagopalan, Erin Pellot, Jonathan L. Koff, Jayne B. Robinson

**Affiliations:** 1 Department of Biology, University of Dayton, Dayton, OH 45469, USA; 2 Department of Chemical and Materials Engineering, University of Dayton, Dayton, OH 45469, USA; 3 Integrated Science and Engineering Center, University of Dayton, Dayton, OH 45469, USA; 4 Section of Pulmonary, Critical Care and Sleep Medicine, Department of Internal Medicine, Yale University School of Medicine, New Haven, CT 06520, USA; 5 School of Medicine, Technical University of Munich, 81675 Munich, Germany

**Keywords:** *Pseudomonas aeruginosa*, porphyrin, phage therapy, phage–antibiotic synergy (PAS), biofilm, murine lung model, cystic fibrosis

## Abstract

*Pseudomonas aeruginosa* (PsA) is an opportunistic bacterial pathogen that causes life-threatening infections in individuals with compromised immune systems and exacerbates health concerns for those with cystic fibrosis (CF). PsA rapidly develops antibiotic resistance; thus, novel therapeutics are urgently needed to effectively combat this pathogen. Previously, we have shown that a novel cationic Zinc (II) porphyrin (ZnPor) has potent bactericidal activity against planktonic and biofilm-associated PsA cells, and disassembles the biofilm matrix via interactions with eDNA In the present study, we report that ZnPor caused a significant decrease in PsA populations in mouse lungs within an in vivo model of PsA pulmonary infection. Additionally, when combined with an obligately lytic phage PEV2, ZnPor at its minimum inhibitory concentration (MIC) displayed synergy against PsA in an established in vitro lung model resulting in greater protection of H441 lung cells versus either treatment alone. Concentrations above the minimum bactericidal concentration (MBC) of ZnPor were not toxic to H441 cells; however, no synergy was observed. This dose-dependent response is likely due to ZnPor’s antiviral activity, reported herein. Together, these findings show the utility of ZnPor alone, and its synergy with PEV2, which could be a tunable combination used in the treatment of antibiotic-resistant infections.

## 1. Introduction

In recent years, resistance to antibiotics has emerged as a significant global health concern due to the severe medical impact that results from resistant pathogens and the limitations of effective treatments [1,2,3,4,5,6,7]. In 2019, the Centers for Disease Control and Prevention reported that more than 2.8 million infections and 35,000 deaths in the United States alone were caused by antibiotic-resistant bacteria [8]. This exponential rise of antibiotic-resistant pathogens, and the increasing degree of human health concerns, warrants the need for alternative antimicrobial strategies [9].

*Pseudomonas aeruginosa* (PsA), is a Gram-negative, opportunistic pathogen linked to severe respiratory, gastrointestinal, and systemic infections in patients with compromised immune systems. PsA infections are also a threat to patients with severe burns, diabetic foot ulcers, and on mechanical ventilation. PsA is responsible for 32,600 infections among hospitalized patients, with 2700 deaths in 2017 [10]. Moreover, approximately 60% of individuals with cystic fibrosis (CF) have respiratory tracts colonized with PsA, translating to decreased lung function and quality of life [11,12]. PsA forms robust biofilms in the lungs in CF, which contributes to these persistent infections [13].

Traditional PsA infections are treated with antibiotics, including the main classes of aminoglycosides, fluoroquinolones, polymyxins, and the β-lactams [14]. These antibiotics function against PsA through a number of mechanisms, including slowing bacterial growth or serving as a bactericidal agent and destroying the cells directly. However, repeated use of antibiotics has been shown to lead to multidrug-resistant PsA, strains which have increased the already significant problem in CF [15]. Beyond antibiotics, other treatment options for PsA infections include anti-virulence agents, which function by disrupting essential host infection mechanisms [14].

PsA’s ability to create biofilms has made it a model organism to study persistent infections such as those in the CF lung, as well as to understand the role of biofilms in the acquisition of antibiotic-resistance genes [16,17,18,19,20]. Biofilms, formed when bacterial cells irreversibly attach to a surface, are comprised of a hydrated matrix primarily composed of exopolysaccharides, proteins, and eDNA [21,22,23,24,25,26]. This matrix provides bacteria protection from antibiotic challenge and tensile support against mechanical disruption. Remarkably, biofilms are reported to exhibit up to a thousand-fold increase in resistance to antibiotics compared to cells growing planktonically [22,27,28,29,30,31]. Due to the close proximity of bacterial cells within biofilms and the subsequent gene transfer that can occur, biofilms frequently develop multidrug-resistant (MDR) strains [20,21,32,33,34].

Given the growing threat of MDR strains, new antibacterial therapies are being investigated, including the use of bacteriophages (phage therapy). Lytic bacteriophages destroy their bacterial host and are effective against recalcitrant and antibiotic-resistant infections [35,36,37]. A number of phages are selectively active against antibiotic-resistant PsA, in both planktonic and biofilm growth [36,37,38,39]. One example is the obligately lytic PEV2 phage, which specifically targets and kills cells of the PsA strain PAO1 [40,41]. We previously reported that PEV2 was not toxic to human lung cells in vitro, and protected the lung cells from PsA, by selectively killing the bacteria [42]. However, there are some limitations to phage therapy, including that the phages are highly host-specific, recent development of host resistance, and limited infection rates in bacterial biofilms due to their mechanical properties.

There is also a move to identify unique small molecules, or therapeutic agents, that can permeate or deconstruct the biofilm matrix. These molecules would allow greater penetration of extant antibiotics and therapies, such as phages. Ideally, these biofilm deconstruction agents would also directly kill the bacterial cells alone and in combination with other therapeutics. Porphyrins are small heterocyclic molecules often found in nature (e.g., heme, chlorophyll, and cytochromes) that mechanistically function by producing high levels of reactive oxygen species (ROS) following photoactivation [43,44,45]. These properties have led to the use of porphyrins as photosensitizers (PS) for photodynamic therapy (PDT) [46,47]. More recently, porphyrins have been studied for their use in antimicrobial photodynamic therapy (aPDT), but this treatment is limited by the need for light activation [21,43].

Previously, we developed a novel cationic, zinc-containing porphyrin that does not require photoactivation to target and destroy Gram-negative and Gram-positive bacteria in the absence of photoactivation, yet retaining its ability for increased potency during photoactivation [21,48]. This porphyrin is (5,10,15-tris (*N*-methyl pyridyl)-20-pentafluorophenyl porphyrinatozincTris-4-methylbenzenesulfonate), a cationic zinc-containing porphyrin that we have named ZnPor (II) [48,49]. We recently provided evidence that ZnPor acts on the PsA biofilm matrix via eDNA and that this mechanism is distinct from its ability to kill PsA cells [21]. Further, pre-treatment of PsA biofilms with ZnPor enhanced the antibiotic activity of the extant antibiotic tobramycin. ZnPor treatment of PsA planktonic cells caused a 4-fold decrease in the minimum inhibitory concentration (MIC) of tobramycin, even without photoactivation [21]. ZnPor has a documented MIC of 4 µg/mL and a minimum bactericidal concentration (MBC) of 8 µg/mL for PsA PAO1 planktonic-grown cells in the absence of light [21,48]. These unique properties and activities make ZnPor a novel and promising antibacterial therapeutic option for PsA and other antibiotic-resistant bacteria [21].

While extant antibiotics and phages have unique antimicrobial effects individually, using them in combination is a promising option for persistent infections [50,51,52]. The goal of phage–antibiotic synergy (PAS) is to use a combination of a phage and an antibiotic to create a synergistic action against pathogenic bacteria, thereby evading or minimizing the development of bacterial resistance. For example, Duplessis showed that when merged with a sub-efficacious dose of meropenem, a combination of phages reduced the bacterial burden of an antibiotic-resistant strain of PsA and decreased mortality in a murine pneumonia model [52].

While ZnPor is not yet classified as an antibiotic, it has potent antibacterial activity, and has the potential to be exceptionally beneficial in situations where PsA has developed antibiotic resistance [21]. Thus, the combination of ZnPor with a phage, such as PEV2, may be considered an example of PAS. In this study, we investigated the ability of a unique porphyrin (ZnPor) to kill PsA cells in vivo using a mouse model of PsA pneumonia [53]. We also examined the use of ZnPor alone, and in combination with the PEV2 phage, against PsA using an established in vitro human pulmonary cell model and within an established biofilm [2]. The results of this study add substantially to our understanding of the effectiveness of ZnPor in killing PsA under a variety of conditions, both in vivo and in vitro, and further confirm ZnPor’s biocompatibility and promise as a novel antibiotic agent.

## 2. Results

### 2.1. ZnPor Decreases Pseudomonas aeruginosa Pulmonary Infection in Mice

**Figure 1:**
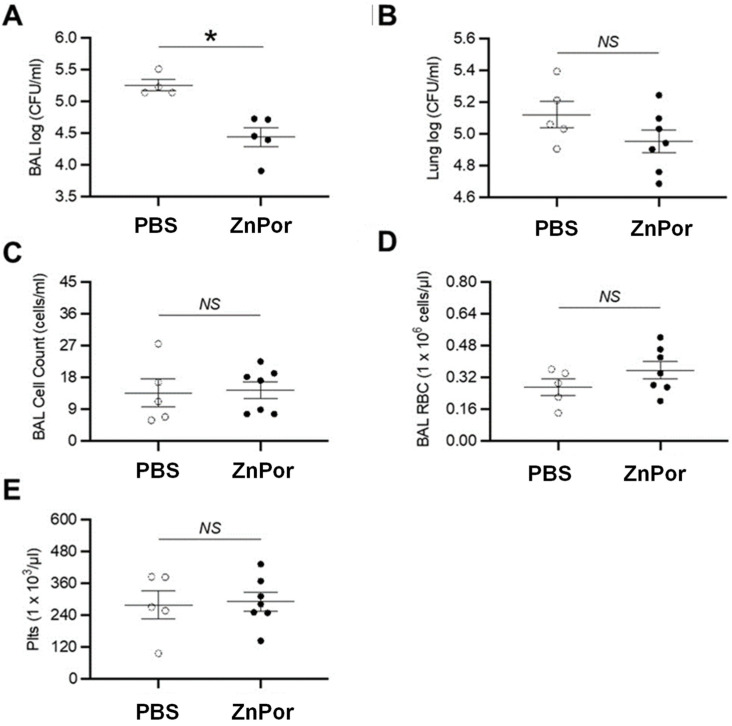
Effects of ZnPor treatment in a mouse model of PsA pulmonary infection. The capacity of ZnPor (12.5 µg delivered) to reduce PsA pulmonary infection was tested in wild-type mice (n = 5–7 mice/group, repeated twice). Bacterial load analysis for (**A**) BAL and (**B**) lung homogenates. Measurements in BAL for (**C**) cell count, (**D**) red blood cells, and (**E**) platelets. * *p* < 0.05, Mann–Whitney U test. BAL, bronchoalveolar lavage; plts, platelets; RBC, red blood cells; *NS*, not significant.

The capacity for ZnPor to decrease PsA pulmonary infection was tested in wild-type mice. Mice were infected with 2.5 × 10^6^ CFU of PsA (strain PAO1), delivered intratracheally via the oral route. After PsA infection, ZnPor (250 µg/mL) or PBS alone (vehicle) was delivered in a similar route. Subsequently, bronchoalveolar lavage (BAL) and lungs were collected at 20 h for quantitative bacteriology, via calculating colony-forming units. ZnPor treatment resulted in a statistically significant reduced BAL bacterial load (Figure 1A). ZnPor treatment also resulted in reduced lung homogenate bacterial load, but this did not reach statistical significance. (Figure 1B). There were no differences in BAL total cell count, red blood cell count, and platelet count (Figure 1C–E), demonstrating that ZnPor did not unintentionally target RBCs or platelets.

### 2.2. ZnPor Reduces PsA Infections of H441 Lung Cells In Vitro

In our previously published work, we determined that ZnPor has an MIC of 4 µg/mL and an MBC of 8 µg/mL against PsA strain PAO1 planktonic cells [21]. Further, we established that biofilm-associated PAO1 cells were killed, and the matrix was disassembled, following ZnPor exposure at the MIC in PBS solution.

**Figure 2:**
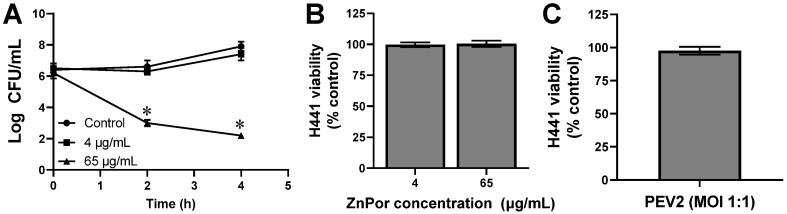
ZnPor concentration-dependent killing of PsA PAO1 cells and independent effects of ZnPor and PEV2 phage on the viability of H441 human lung cells. (**A**) Without photoactivation, 65 µg/mL ZnPor killed PsA cells with greater than a 3-log reduction. At the MIC (4 µg/mL), no change to PAO1 levels were identified (**B**) H441 cell viability was not significantly reduced by ZnPor at either 4 or 65 µg/mL following a 24 h exposure. (**C**) H441 cell viability was unaffected by the addition of a PEV2 phage at an MOI of 1:1 following 24 h exposure. Neither ZnPor nor PEV2 displays any statistically significant toxicity against H441 lung cells. * indicates statistical significance from untreated control (*p* < 0.05, n = 3).

In contrast, mammalian pulmonary cells are typically maintained in a cell culture medium with a high protein and an ionic content of 150 mM which has been shown to induce porphyrin self-aggregation [54]. Therefore, it was necessary to determine the efficacy of ZnPor in RPMI 1640, which we used to culture H441 pulmonary cells in this study. Figure 2A shows the antibacterial activity of ZnPor in RPMI. The higher concentration of 65 µg/mL demonstrated greater than a 3-log reduction in PAO1 within 4 h. Previously we have shown complete killing by 7 h in a minimal salt medium supplemented with glucose [21]. In contrast, no significant changes in bacterial cell concentrations were noted at 4 µg/mL concentration, which confirms the MIC we observed in the MSG medium.

We assessed the toxic effects of ZnPor and PEV2 alone on the human lung cell line H441. H441 cells were exposed to either 4 µg/mL ZnPor (MIC) or 65 µg/mL (8X the MBC) for 24 h [21]. As shown in Figure 2B, neither concentration of ZnPor caused a reduction in H441 cell viability. The effect of PEV2 without the presence of PsA was evaluated at 24 h after adding 84 PFU/mL to H441 cells, as seen in Figure 2C. This number of phages is the same as added in the trials where PsA cells were present and represents an MOI (multiplicity of infection) of 1:1 for PEV2:PsA. PEV2 did not reduce H441 cell viability by 24 h post-addition, which is in agreement with our prior studies that found PEV2 was not toxic to A549 lung cells [42]. These results indicate that both ZnPor and the PEV2 phage are biocompatible with the conditions used in the present study.

### 2.3. ZnPor and PEV2 Combined Exhibit a Positive Synergy against PsA Infected H441 Cells in an In Vitro Model

A PsA–H441 infection model was established (see Materials and Methods), and the effects of ZnPor or PEV2 alone and in combination were tested against PsA PAO1 cells. Initial experiments were conducted in 96-well plates at an MOI of 10,000:1 (H441 to PAO1 cells). H441 cells were infected with PsA PAO1 cells at this concentration to allow for the evaluation of a longer infection time. The denoted mixtures of PAO1, ZnPor, and PEV2, were added at the same time to H441 lung cells, with ZnPor and PEV2 concentrations increasing in a checkerboard manner; row and column increasing concentrations of either ZnPor, PEV2, or both. ZnPor was evaluated at concentrations of 0, 4, 33, and 130 µg/mL. The PEV2 phage was added at an MOI of 0, 1:1, 10:1, or 100:1. The plates were incubated at 37 °C for 24 h followed by an assessment of PAO1 cell populations (CFU/mL).

**Figure 3:**
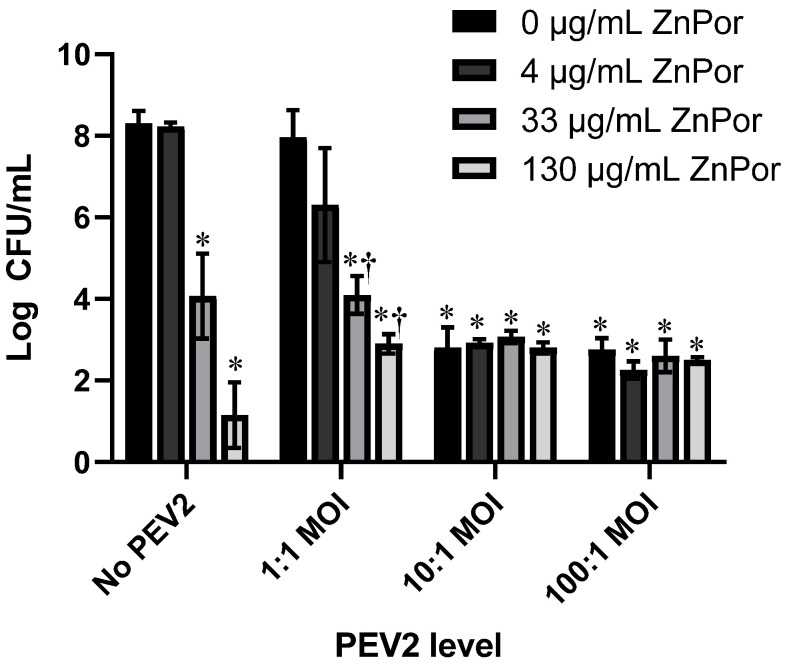
Assessment of ZnPor/PEV2 combinatorial effects against PsA PAO1 cells within 96-well plates. H441 cells were infected with PsA cells at an MOI of 10,000:1 (H441 to PAO1 cells) and dosed with either ZnPor or PEV2 alone, as well as ZnPor in combination with the PEV2 bacteriophage. PAO1 viability was measured after 24 h using the viable plate count assay (CFU per mL). * Indicates significance from PsA control; and † indicates statistical significance between PEV2 alone and ZnPor + PEV2 treatment at the same MOI (*p* < 0.05, n = 3). CFU/mL, colony-forming units/mL; MOI, multiplicity of infection.

ZnPor alone decreased PsA populations in a dose-dependent manner, as shown in Figure 3. At the highest concentration (130 µg/mL), there was over a 6-log reduction in PsA CFU/mL, while there was no reduction at 4 µg/mL. This confirms the robust antibacterial activity we reported previously [21]. There was over a 1.5-log decrease in PsA CFU/mL when ZnPor (4 µg/mL) and PEV2 (MOI 1:1) were added in combination compared to the PEV2-only control, indicating synergy, but the difference was not statistically significant. At higher MOIs of PEV2 (10:1 and 100:1), the additive effect disappeared and failed to achieve the killing of PsA by ZnPor alone at 130 µg/mL. The aim of this initial matrix evaluation was to determine the lowest possible effective concentration of ZnPor to be used in combination with PEV2 where noticeable PsA killing was observed. These data suggest that at its MIC, ZnPor, while not bactericidal, can enhance the killing of PsA cells by the lytic phage PEV2 [21]. The proposed mechanism for this effect can be found in the Discussion.

### 2.4. Effect of ZnPor and PEV2 Treatment on the Viability of H441 Lung Cells Infected by PsA

The concentrations of ZnPor and PEV2 that showed promise against PsA populations shown in Figure 3 were further investigated. Therefore, we next measured H441 viability within an infection model following treatment combinations of ZnPor and PEV2. For these experiments. H441 cells were grown in 6-well plates to provide larger working volumes and cell counts. The experimental dosages were 4 µg/mL and 65 µg/mL ZnPor with PEV2 at 1:1 MOI. Neither ZnPor dosage impacted H441 viability, as shown in Figure 2B.

**Figure 4:**
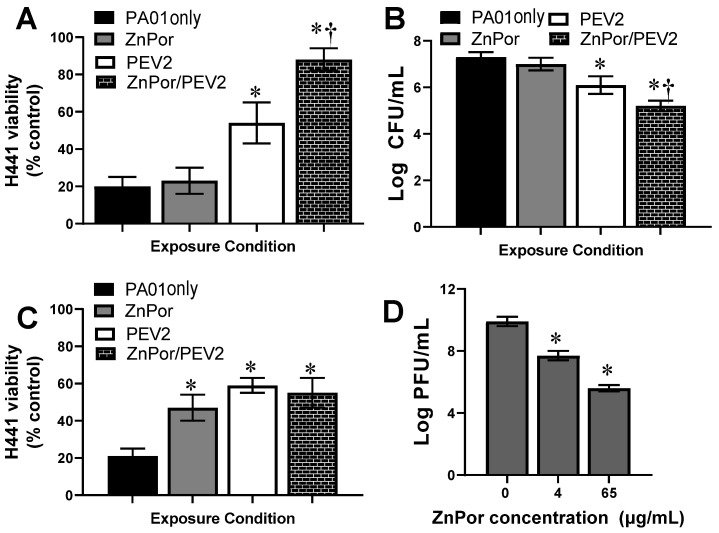
Effect of ZnPor, PEV2, and both on H441 lung cell viability and reduction of PsA PAO1 cell populations in vitro. Following establishment of PsA–H441 infections, cells were supplemented with (1) ZnPor alone at 4 or 65 µg/mL, (2) PEV2 alone (MOI 1:1), or (3) both ZnPor and PEV2. (**A**) H441 cell viability was measured after 24 h for all experimental conditions with 4 µg/mL ZnPor. (**B**) PsA bacterial viability was assessed after 24 h exposure to ZnPor (4 µg/mL), PEV2 (1:1 MOI), or both concurrently by measuring CFU/mL. (**C**) H441 cell viability was measured after 24 h for all experimental conditions with a ZnPor dosage of 65 µg/mL. (**D**) Following the establishment of an H441-PsA infection, ZnPor was added at either 4 or 65 µg/mL and PEV2 at 84 PFU/mL, and incubated for 24 h. PFU/mL levels decreased significantly at both ZnPor concentrations, indicating that ZnPor was reducing PEV2 activity. * Comparison to untreated control; † comparison of PEV2 and PEV2/ZnPor treatment (*p* < 0.05, n = 3). CFU/mL, colony-forming units/mL; MOI, multiplicity of infection; PFU/mL, plaque-forming units/mL.

Treatment of H441 cells with PsA alone resulted in an 80% loss of viability (Figure 4A). Only at 4 µg/mL did ZnPor not rescue H441-PsA-infected cells, as evidenced by the low viability of H441 cells. This is not unexpected as ZnPor at this concentration is not bactericidal against PsA populations (Figure 3). Compared to ZnPor and PEV2 alone, PEV2 combined with ZnPor at 4 µg/mL exhibited a statistically significant rescue of H441 cell populations. ZnPor combined with PEV2 resulted in approximately 90% H441 cell viability compared to untreated controls (Figure 4A). Looking at bacterial loads, PsA CFU/mL were not decreased by ZnPor at 4 µg/mL, as expected; however, PEV2 combined with ZnPor resulted in a statistically significant reduction in CFU/mL of PsA (Figure 4B).

Looking specifically at the H441 viability (Figure 4A) and the bacterial levels in the in vitro system (Figure 4B), we sought to determine if these responses were exhibiting a synergistic effect. Table 1 shows the numerical values for the increase in H441 viability over the PAO1 control and the decrease in PsA levels for ZnPor (4 µg/mL) or PEV2 (1:1) alone and combination ZnPor/PEV2 conditions. This data demonstrates that for both H441 viability and PsA levels, concurrent treatment with ZnPor and PEV2 resulted in synergistic protection as the observed changes are greater than the sum of the individual treatments.

Figure 4C are the results of our testing of the effect of ZnPor at 65 µg/mL, PEV2 at MOI of 1:1, and the combination of the two. A concentration of 65 µg/mL ZnPor was selected based on Figure 2B and is well above the MBC for PsA [21]. ZnPor at 65 µg/mL resulted in increased H441 cell viability compared to PsA alone, as did PEV2 alone. However, there was no apparent synergy for ZnPor combined with PEV2 at this higher concentration of ZnPor. Further, the protective effect of PEV2 alone seen in Figure 4A was lost. This led us to question whether ZnPor at 65 µg/mL was deactivating PEV2.

To determine whether ZnPor was affecting PEV2 infectivity (Figure 4C), we measured the PFU/mL of PEV2 after exposure to ZnPor. The supernatant from H441 cells infected with PsA PAO1 exposed to ZnPor, and PEV2 was collected, and the PFU/mL was determined. As seen in Figure 4D, PEV2 PFU/mL decreased in a dose-dependent fashion with respect to ZnPor concentration, confirming that ZnPor has viricidal activity. This is the first report we are aware of showing that a porphyrin has antiviral activity in the absence of light activation.

### 2.5. The Effect of ZnPor and PEV2 on PsA Biofilms Grown on Polyethylene

Neither the mouse in vivo nor the H441 in vitro trials addressed biofilm-associated or persistent infections. We previously published a method to test the effectiveness of both ZnPor and other antibiotics on PsA biofilms. In this biofilm protocol, we showed that ZnPor kills individual PsA cells in the biofilm while also deconstructing the matrix [21]. ZnPor can rapidly diffuse throughout the biofilm, which translates to the active deconstruction of the matrix via eDNA and simultaneously renders the biofilm more porous [21]. This led us to test whether ZnPor enhanced the ability of phages to access and destroy PsA bacteria inside a biofilm matrix.

**Figure 5:**
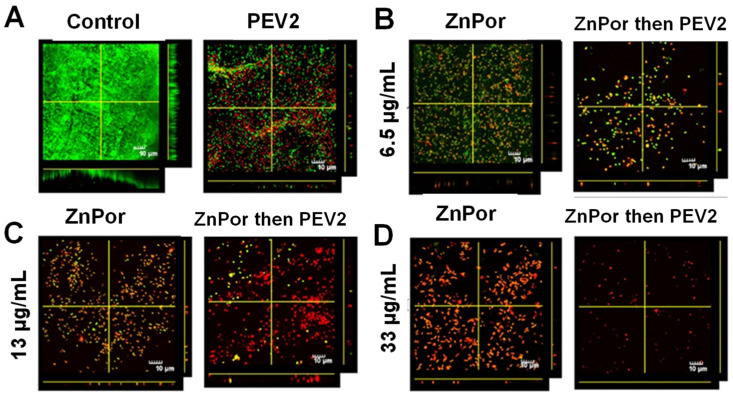
Biofilms of PAO1 treated with ZnPor or PEV2 alone, and in combination in the absence of photoactivation. Biofilms of strain PAO1 were grown on coupons cut from polyethylene in a CDC-approved bioreactor. The biofilms were then exposed to (**A**) PBS (control) or PEV2 (MOI 10:1), or ZnPor and ZnPor/PEV2 exposure at ZnPor concentrations of (**B**) 6.5 µg/mL, (**C**) 13 µg/mL, or (**D**) 33 µg/mL. For joint ZnPor/PEV2 experiments, biofilms were pre-treated with ZnPor for 2 h followed by the addition of phage PEV2 (MOI 10:1) and incubated for 4 h. Biofilms were stained with LIVE/DEAD ^TM^ fluorescent stain and imaged using confocal laser scanning electron microscopy (CLSM) at 60×. Green = LIVE, red = DEAD. Control refers to a biofilm that received no treatment.

Using this previously established protocol, a PsA biofilm was developed on polyethylene substrates. These substrates then underwent a matrix of experimental conditions, including combinations of ZnPor and PEV2, at varying ZnPor concentrations. In these experiments, the biofilms were pre-treated with ZnPor, which is able to permeate and deconstruct the matrix of the biofilm. The latter addition of PEV2 was then able to directly diffuse into the biofilm and selectively destroy PsA cells. Figure 5A shows that in biofilms treated with PEV2 alone, dead cells are present; however, the biofilm remains dense and intact. In contrast, the introduction of ZnPor shows a concentration-dependent killing of PsA cells, along with a corresponding loss of biofilm structure (Figure 5B–D). At higher ZnPor doses, the biofilm was reduced to a monolayer of PsA cells, which were predominantly dead. Looking at combined treatments, at all concentrations of ZnPor, the addition of PEV2 resulted in greater numbers of dead cells and further loss of biofilm structure. It can be qualitatively determined that synergy is occurring in Figure 5B, where at the lowest ZnPor dose with PEV2, there is a significant reduction in the number and density of live PsA cells compared to ZnPor or PEV2 alone.

## 3. Discussion

While antibiotics are still the most common tool used to treat bacterial infections, the rise of antibiotic-resistant pathogens has spurred the search for alternative methods of treatment [21,55]. PsA is known for its ability to rapidly acquire antibiotic resistance both intrinsically through efflux pumps and externally through the production of biofilms [56]. Thus, there is an urgent need for novel therapeutics, especially small molecules that lead to the degradation of the biofilm matrix.

ZnPor is a cationic porphyrin that has bactericidal activity against both Gram-negative and Gram-positive bacteria. Using PsA as a model organism, we have established that ZnPor acts to deconstruct the biofilm matrix of PsA via interactions with eDNA [21]. Unlike other porphyrins being assessed for antimicrobial activity, ZnPor’s bactericidal activity and ability to deconstruct the biofilm matrix do not require light activation. Although novel ways are being developed to bring light of different wavelengths and modes to the interior parts of the human body, ZnPor provides a potential solution to a major limitation of using porphyrins in aPDT by eliminating the need to provide light for activity. The killing of planktonic and biofilm-associated cells is distinct from its action on the biofilm matrix itself; however, the combined activities are of particular value in the treatment of pathogenic bacteria known to form biofilms, such as persistent and recurrent PsA infections in CF patients. Further, ZnPor has been shown to be non-toxic to both *Drosophila melanogaster* and lung cells, demonstrating its potential as a biomedical therapeutic [47].

A valuable finding in this study is that ZnPor was shown to decrease the PsA load in a murine model of pulmonary infection (Figure 1). Importantly, the mice treated with ZnPor did not exhibit signs of overt toxicity, as assessed by no alterations to BAL total, RBC, and platelet counts (Figure 1C–E). This last finding suggests that ZnPor could be employed in the presence of RBCs and platelets, which was a novel discovery for this experimental porphyrin. We did not test the ability of ZnPor to reduce bacterial load when there was a longer duration (i.e., >30 min) between PsA infection and ZnPor administration, but this would be a logical next step to explore in a future study. Further, administration of multiple doses is likely to further reduce PsA populations. Interestingly, the volume of ZnPor solution delivered to the mouse resulted in a 10-fold lower number of ZnPor molecules per bacterial cell than in the in vitro assays. This is evidence that higher concentrations may prove more effective at killing PsA in the mouse lung without a corresponding increase in toxicity.

Next, we examined the combinatorial effects of ZnPor and the lytic phage, PEV2, against PsA PAO1 using an in vitro H441 lung cell infection model [42]. As shown in Figure 2B,C, there was no loss of H441 cell viability for any experimental concentration of ZnPor and PEV2. In agreement with previous publications, ZnPor reduced PsA populations in a dose-dependent manner in the absence of the PEV2 phage. At the highest concentration of ZnPor (130 µg/mL), there was over a 5.5-log reduction in PsA CFU/mL. In contrast, there was no difference in CFU/mL when treated with ZnPor at 4 µg/mL (MIC). ZnPor combined with PEV2 resulted in statistically significant synergy both in killing PsA cells and in the protection of H441 viability, demonstrating the presence of a unique synergistic effect (Table 1). Specifically, ZnPor at 4 µg/mL with PEV2 at an MOI of 1:1 resulted in greater killing of PsA cells and thus a dependent increase in H441 viability than observed with either treatment alone (Figure 4A,B). While porphyrins are generally considered non-toxic without photoactivation, the discovery that ZnPor at its MIC enhanced the killing of PsA by the PEV2 phage could be of high value in phage therapy.

We report here for the first time a porphyrin that has antiviral activity in the absence of photoactivation. The data in Figure 4C led us to question whether ZnPor at 65 µg/mL was disrupting phage activity against PsA. Using the lytic PsA phage PEV2, we tested the ability of our novel, patented porphyrin ZnPor to reduce infective competent numbers of PEV2 by measuring PFU/mL (Figure 4D). These results determined that with increased ZnPor concentrations, PEV2 levels were correspondingly decreased. As the PEV2 phage is a DS DNA virus, its replication would be subject to agents that intercalate into DS DNA such as porphyrins.

Previously, we have shown that ZnPor can permeabilize bacterial cells at 4 µg/mL [21]. Thus, we hypothesize that ZnPor generates pores in the cell membrane whereby phage virions may enter the cell without the need to attach to their surface receptor. This supports the PAS evident in Figure 4A,B, in which the joint use of ZnPor and PEV2 synergistically rescued H441 cells from PsA infection. This synergy is only seen at low concentrations of ZnPor because at higher ZnPor concentrations, PEV2 PFU/mL is reduced (Figure 4D). At low ZnPor dosages, this synergy with PEV2 was visualized in PsA biofilms, where the combination of these therapeutics resulted in the biofilm being reduced to a monolayer of mostly dead bacterial cells. Since synergy is evident at the low concentrations, these results indicate that there exists a stoichiometry-based synergy between ZnPor and PEV2 levels. It is important to note that in the biofilm trials ZnPor is added and pre-incubated for 2h in order to deconstruct the matrix prior to the addition of phage. In the case of planktonic cells pre-incubation with ZnPor may also prevent killing of PEV2. Further investigation is warranted for the development of an optimal timing and therapeutic combination. These initial data support that ZnPor and PEV2, in combination, may be useful as an alternative to antibiotic treatment in the lung.

In conclusion, ZnPor and PEV2 independently showed no toxicity towards human epithelial lung cells, and ZnPor reduced pulmonary infection in an in vitro human pulmonary cell model. ZnPor also demonstrated effectiveness against pulmonary PsA infection in mice, demonstrating that ZnPor efficacy successfully bridged the in vitro–in vivo gap. ZnPor was synergistic with the phage PEV2 against PsA infection in human epithelial lung cells at low doses, warranting further study into these findings. In addition to lung infections in CF, PsA strains are primary members of ventilator-associated pneumonia (VAP) lung infections, which are due to complications associated with COVID-19 [57]. Due to its water-soluble nature and biocompatibility, ZnPor could be easily administered via a nebulizer directly into infected lungs with the potential to assist in a number of lung-based infections and diseases upon further investigation.

## 4. Materials and Methods

### 4.1. Preparation of Pseudomonas aeruginosa Culture for Mouse Model

PsA strain PAO1 was cultured by plating the glycerol stock on LB plates [58]. A single colony was picked and grown overnight in LB broth. On the second day, the bacteria were sub-cultured for 1 h to bring them to the linear growth phase. The number of bacteria were estimated by measuring the optical density at 600 nm. The numbers were confirmed by using standard colony-forming units by plating the inoculum on agar plates.

### 4.2. Mouse Model

Mice were bred at the Yale Animal Resource Center at Yale University in specific pathogen-free conditions. Mice were housed in microisolator cages and received food and water ad libitum. All experimental protocols (Protocol# 2021-20025 and EG00054046) were done in accordance with approved guidelines, regulations, and protocols as determined by the Institutional Animal Care and Use Committee at Yale. C57BL/6 mice were purchased from The Jackson Laboratory and bred in-house. Mice 6–10 weeks of age, male and female, were used for experiments. All animals were age- and sex-matched and then randomized into different groups.

### 4.3. Pseudomonas aeruginosa Pulmonary Infection

Mice were anesthetized with isoflurane. Subsequently, 2.5 × 10^6^ CFU of PsA bacteria (strain PAO1) per mouse in 50 µL of PBS was delivered intratracheally via the oral route. Mice were allowed to recover from anesthesia. The mice were anesthetized again with isoflurane, 30 min after the inoculation of bacteria, and ZnPor (250 µM) diluted in 50 µL PBS or 50 µL PBS alone was delivered intratracheally via the oral route. After 20 h, bronchoalveolar lavage (BAL) and lungs were isolated for analysis [59]. For BAL, the trachea was cannulated and perfused with two aliquots of 0.9% cold saline. BAL fluid total cell count, red blood cell count, and platelet count were determined using an automated COULTER cell counter (Beckman Coulter, Brea, CA, USA).

### 4.4. Quantitative Bacteriology

Viable bacterial counts in BAL and lungs were determined using a colony-forming unit (CFU) assay [60]. Serial dilutions of BAL and lung homogenates, respectively, were plated on *Pseudomonas* isolation agar. Bacterial culture plates were incubated overnight at 37 °C. On the second day, bacterial load was calculated by quantifying viable bacterial colonies.

### 4.5. Mouse Model Statistical Analysis

For animal experiments, data were examined by analysis of variance to estimate means and standard errors. Statistical significance was assessed by Mann–Whitney tests. For animal experiments, three independent experiments were performed. Graphs and statistical analysis were completed using GraphPad Prism (version 9.4.1, La Jolla, CA, USA).

### 4.6. Strain Isolation and Maintenance

Cultures of wild-type (PAO1) *Pseudomonas aeruginosa* (PsA) were generously provided by Dr. Eb Pesci (East Carolina University). PsA cultures were streaked onto Luria broth (LB) agar (1%) plates and grown overnight at 37 °C. A single, isolated colony was used to inoculate an overnight culture grown aerobically with shaking at 37 °C in minimal salts and glucose (MSG) media. The concentration of PsA cells used for experimentation was determined through turbidity assessment.

### 4.7. Zinc-(II)-Containing Porphyrin (ZnPor)

The 5,10,15-tris (*N*-methyl pyridyl)-20-pentafluorophenyl porphyrinatozincTris-4-methylbenzenesulfonate) (ZnPor) is a novel zinc-containing porphyrin compound created by Shawn Swavey (University of Dayton, US Patent #9364537). This porphyrin, which has shown exceptional promise, has been extensively studied by our laboratories and is well characterized in prior reports [21,48]. To maintain consistency, Frontier Scientific was contracted to make large ZnPor batches supplied in powder form. Fresh stocks were made weekly, sterilized via filtration using a 0.2 µM filter, and stored at 4 °C and in the dark to maintain porphyrin activity.

### 4.8. Bacteriophage Preparation

PEV2, a lytic bacteriophage targeting PsA, was specifically chosen for this study. The original stock of PEV2 phages was acquired from Dr. Bob Blasdel. PEV2 phage stocks were amplified using the plate lysate method with a soft agar overlay [42]. Briefly, 100 µL of fresh PAO1 stock and 100 µL of 10^7^ PEV2 stock (diluted in phage buffer) were combined in 4 mL of LB media with 0.7% agar. This solution was then mixed and poured onto a 1% LB agar plate and incubated at 37 °C for 24 h. The plates that contained clear and confluent plaques were chosen for harvesting, during which 3 mL of sterile LB broth was added, and the soft overlay was removed. The harvested suspension was vortexed and incubated on ice for 30 min, then centrifuged at 10,000 rpm for 10 min at 4 °C. The supernatant contained the concentrated crude phage and was filter-sterilized using a 0.2 µM filter. Contents were tested for contamination, underwent tittering to determine phage stock concentrations, and were stored in a sterile phage buffer at 4 °C.

### 4.9. Tissue Culture Maintenance

Human lung cells, H441, obtained from a papillary adenocarcinoma, were purchased from American Type Culture Collection. H441 cells were grown in RPMI 1640 media supplemented with 10% fetal bovine serum and 1% penicillin/streptomycin and stored in a humidified incubator maintained at 37 °C and 5% CO_2_. For all experimentation, confluent H441 cultures were washed and removed from the culture dishes and plated at the denoted conditions in RPMI 1640 media without FBS or antibiotics.

### 4.10. ZnPor Activity against PsA in RPMI Media

An overnight culture of planktonic PAO1 cells was diluted to 0.15 OD (600 nm), which was subsequently diluted 100-fold to give ~10^6^ CFU/mL in RPMI media without FBS or antibiotics. PsA cells were dosed with fresh ZnPor to a final concentration of 0, 4, or 65 µg/mL and incubated at 37 °C. At predetermined times, colony-forming units (CFU/mL) were determined within each culture using serial dilution and plating to evaluate bacterial concentrations. Data is included from three independent experiments.

### 4.11. ZnPor and PEV2 Biocompatibility in H441 Cells

H441 cells were seeded into 6-well plates at a concentration of 8.4 × 10^5^ cells/mL and incubated overnight to allow cells to adhere. The next morning, cells were washed and replenished with fresh media, without antibiotics or serum, including the denoted experimental conditions. For ZnPor biocompatibility assessment, the experimental porphyrin was added to the cells at a concentration of 4 or 65 µg/mL. For phage evaluation, H441 cultures were exposed to PEV2 at a concentration that would represent a 1:1 MOI (PsA: phage) in the infection model. All experimental conditions were incubated for 24 h, after which the cells were washed, removed from the cell culture plate via trypsin, and counted using an Invitrogen Countess Cell Counter. Cell viability was determined by comparing experimental conditions against an untreated H441 control. Data is included from three independent experiments.

### 4.12. Establishment of the PsA–H441 Infection Model

Prior to the introduction of ZnPor or PEV2, a joint bacterial and mammalian culture needed to be established. Experimentation was planned for a 96-well and 6-well plate format, so the model needed to be optimized to function effectively in both environments while maintaining consistent exposure conditions. The base exposure media was RPMI 1640 without antibiotics or FBS. Cell densities for both H441 and PsA were optimized to ensure that the cells could co-exist and remain viable during the experimental time frame of 24 h. It was determined that the H441 cells should be infected with PsA at a multiplicity of infection (MOI) of 10,000:1 (H441 cells to bacteria). For 96-well and 6-well plates, cells were seeded at 2.4 × 10^5^ and 8.4 × 10^5^ cells/mL, respectively.

### 4.13. Testing of ZnPor/PEV2 Combinations In Vitro

H441 tissue cells were seeded into 96-well plates and incubated overnight. Tissue cells were then infected with PsA at an MOI of 10,000:1 (H441: PsA). Three experimental conditions of ZnPor were utilized, 4, 33, and 130 µg/mL, representing low, medium, and high dosages, respectively. Several variations of PEV2 levels were incorporated into the experimental matrix, to create final MOI levels (phage: PsA) of 1:1, 10:1, and 100:1. Untreated and PsA-only controls were also included in the experimental design. After the H441 cells were dosed with the denoted ZnPor/PEV2 combination, they were incubated for 24 h. At the end of the exposure, the media was removed and underwent colony-forming units (CFU) evaluation via serial dilution and plating to determine bacterial load levels. Data is included from three independent experiments.

### 4.14. H441 Viability of H441 Cells Following ZnPor and PEV2 Co-Treatment

H441 lung cells were seeded into a tissue-culture-treated 6-well plate at 8.4 × 10^5^ cells/mL and incubated overnight. A mix of PAO1 cells, ZnPor, and PEV2 was added at the same time to H441 lung cells. An overnight culture of planktonic PAO1 cells was grown at 37 °C in MSG with shaking, then diluted to produce a ratio of H441:PAO1 of 10,000:1 (84 cells of PAO1 per mL). The PEV2 phages were added at an MOI of 1:1 to PAO1 cells (MOI of the phage was based on the matrix experiments). Experimental concentrations of ZnPor selected were 4 and 65 µg/mL, based on the matrix results and prior studies of ZnPor [21]. The mix of PsA, PEV2, and ZnPor was added to the H441 cultures in the absence of serum proteins and antibiotics and was incubated for 24 h. At the end of the exposure duration, the cells were washed twice, removed via trypsin, and counted through a trypan blue assay using an Invitrogen Countess Cell Counter. All experimental conditions were compared to an untreated control to determine H441 viability levels. Treatments were done in triplicate with three independent experiments.

### 4.15. Effect of ZnPor on PEV2 Phage Populations

To test whether ZnPor inactivates PEV2, we used the same conditions outlined in 4.14. A PsA–H441 infection was established in 6-well plates. The mix of PAO1 cells, ZnPor, and PEV2 was added at the same to H441 lung cells and incubated for 24 h. Following the 24 h exposure, the PEV2-containing supernatant was collected. PEV2 activity levels were determined by a PFU analysis, carried through serial dilution and a soft agar overlay procedure as described in 4.8. Treatments were done in triplicate with three independent experiments.

### 4.16. PsA Biofilms Development on Polyethylene and Viability Assessment

CDC-approved bioreactors containing polyethylene coupons were inoculated with 0.15 OD (600 nm) of an overnight planktonic culture of PAO1 in MSG media and grown for ~16–18 h at 37˚C and 50 RPM in order to create a PsA biofilm. Following ~16–18 h incubation, the overnight media was changed out for 150 mL of PBS and the biofilm-covered coupons then underwent exposure to the denoted experimental conditions of: (1) an untreated control; (2) ZnPor at concentrations of (6.5 µg/mL, 13 µg/mL, and 33 µg/mL); (3) phage PEV2 at an MOI of 10:1 (phage to PAO1 cells); (4) a combined treatment of ZnPor followed by PEV2. Biofilms treated with ZnPor were incubated for 2 h, and biofilms treated with phage PEV2 were incubated for 4 h. Biofilms subjected to the combined treatment were incubated with ZnPor for 2 h, followed by a treatment of phage PEV2 added directly to the media for 4 h. As the treatments were added to the bioreactor, RPM was increased to 70 RPM.

In order to assess PsA viability and to visualize the biofilm after exposure to the experimental conditions, the polyethylene coupons were removed from the bioreactors, gently washed in distilled water, and treated with LIVE/DEAD ^TM^ stain, which is a well-established protocol to assess and visualize bacterial density [61]. During experimentation, the LIVE/DEAD stain protocol was followed as per the manufacturer’s recommendations. All coupons were imaged using confocal laser scanning electron microscopy (CLSM) at 60×. Green = LIVE, red = DEAD. Control refers to a biofilm that received no treatment.

### 4.17. Statistical Analysis

Data are expressed as the mean ± standard error of the mean (SEM). Statistical significance was determined through either a one-way or two-way ANOVA with a Bonferroni post-test using GraphPad Prism. For all experimentation, a minimum of three independent trials were performed. The threshold for statistical significance was a *p*-value less than 0.05 and indicated with an asterisk (*).

## Figures and Tables

**Table 1:** Determination of Synergistic Behavior following 4 µg/mL ZnPor and PEV2 Co-Treatment.

	Increase in H441 Viability (%)	Decrease in PsA CFU/mL (log)
ZnPor	2.4	0.3
PEV2	33.4	1.2
ZnPor/PEV2	67.8	2.1

## Data Availability

Not applicable.
